# Heat-Initiated Chemical Functionalization of Graphene

**DOI:** 10.1038/srep20034

**Published:** 2016-01-28

**Authors:** Guodong Gao, Dandan Liu, Shangcheng Tang, Can Huang, Mengci He, Yu Guo, Xiudong Sun, Bo Gao

**Affiliations:** 1 Institute of Modern Optics, Key Lab of Micro-optics and Photonic Technology of Heilongjiang Province, Department of Physics, Harbin Institute of Technology, Harbin 150001, China; 2 School of Materials Science and Technology, Harbin Institute of Technology, Harbin 150001, China

## Abstract

A heat-initiated chemical reaction was developed to functionalize CVD-grown graphene at wafer scale and the reaction was universally extended to carbon nanotubes, and other precursors that could be thermally converted to active radicals. The chemical reaction can occur in absence of oxygen and water vapor when the temperature is above the decomposition temperature of the reactants. The chemical reaction was also found to be substrate-dependent due to surface doping and inhomogeneity. A large-scale graphene pattern was demonstrated by combing with microfluidic technique. This heat-initiated solid-phase chemical reaction provides a facile and environmentally friendly approach to functionalize carbon nanomaterials with various functional groups.

Graphene is a two-dimensional, atomically thin lattice of sp^2^ bonded carbon atoms and has remarkable electronic, mechanical, optical and thermal properties^1^. Modifying the basic properties of pristine graphene is crucial for incorporating graphene into a variety of applications. For example, in pristine graphene the valence band and the conduction band touch at the Fermi level at K (K’) point of Brillouin zone, making graphene not be able to be switched off. Therefore it needs to open a band gap for the application of graphene in field-effect transistors (FETs) at wafer scale^2^. Chemical functionalization^3^,^4^,^5^, which changes the hybridization of graphitic atoms from sp^2^ to sp^3^, has been shown to be effective in introducing a band gap into graphene^6^,^7^,^8^,^9^,^10^ and metallic single-walled carbon nanotubes (SWNTs)^11^,^12^. Chemically functionalized graphene is also of great importance for many other applications beyond electronics. E.g., the transparency and adhesion of conductive graphene films for touch screens^13^, the efficiency of graphene-based biosensors^14^,^15^, the capacitance and cyclic lifetime of supercapacitor electrodes^16^,^17^,^18^, as well as the biocompatibility and cytotoxicity of graphene^19^,^20^ can be improved by controlling the surface functional groups of graphene.

However, pristine graphene is one of the most chemically inert materials because high energy barriers need to be overcome due to the rigid planar structure and remarkable intarlayer conjugation^21^. The chemical functionalization of graphene to create derivatives with various structures and tunable properties presents an exciting new direction in both theoretical and experimental research. By diazonium chemistry^8^,^22^ and photochemistry^7^,^23^,^24^ various functional groups have been grafted onto graphene. For the diazonium chemistry, stirring-assisted solution reaction is tedious and is often not preferred by those who are not expert in chemistry. For the photochemistry, either a tiny focused laser spot is used for enough high intensity^23^,^24^, resulting in a localized functionalization and a low-efficiency method, or deadly chlorine is used^7^. Therefore, facile and environmentally friendly approaches for the chemical functionalization of graphene to tailor its electronic, optical, and chemical properties are still highly desirable.

In this article, a heat-initiated chemical reaction was developed to functionalize chemical vapor deposition (CVD)-grown graphene at wafer scale. Raman and X-ray photoelectron spectroscopy (XPS) measurement showed that the absorbed benzoyl peroxide (BPO) molecules on graphene decomposed above 75 °C to active radicals, which further reacted with directly adhered graphene in nitrogen atmosphere. The chemical reaction is highly sensitive to temperature, oxygen and water vapor. The chemical functionalization could be controlled by heating time and be tuned by substrate via surface doping and inhomogeneity. It is found that this heat-initiated solid-phase chemical reaction could be applied to carbon nanotubes and other molecules that can be thermally converted to active radicals. A large-scale graphene pattern was also demonstrated by combing with microfluidic technique.

## Results

### Heat-initiated chemical functionalization of graphene

**Figure 1:**
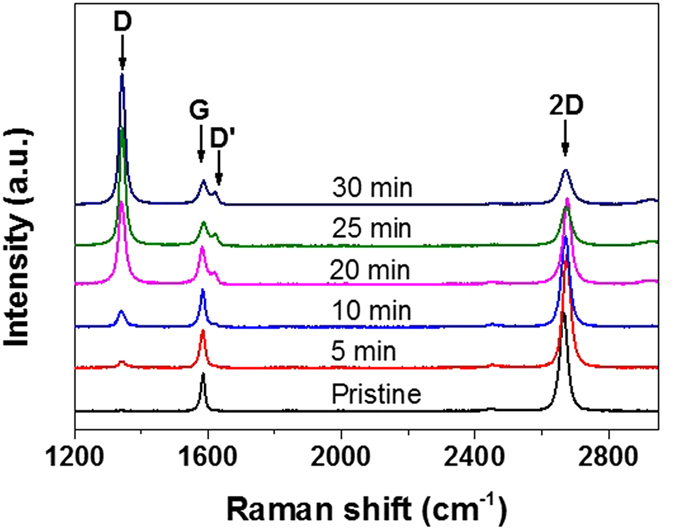
Raman spectra of pristine graphene and BPO-functionalized graphene heated at 80 °C for 5 min, 10 min, 20 min, 25 min and 30 min in nitrogen atmosphere, respectively.

Single-layer graphene films were grown on 25 μm-thick copper foil using low-pressure CVD. Unless stated, for subsequent characterizations and functionalization, graphene was transferred onto SiO_2_ substrate via polymethyl methacrylate (PMMA)-mediated method. Single-layer graphene was identified by micro-Raman spectroscopy equipped with a 532 nm laser line. As shown in Fig. 1, Raman spectra of pristine graphene have two characteristic peaks, the G band which is due to the E_2g_ vibrational mode of sp^2^ bonded carbon atoms and is observed at ~1580 cm^−1^, and the 2D band at ~2670 cm^−1^ which is a second-order vibration caused by the scattering of phonons at the zone boundary^25^. The intensity of the 2D band is about 2–3 times the intensity of the G band, indicating a single-layer graphene.

The pristine graphene samples were immersed into a 10 mM acetone solution of BPO for 30 min and were then taken out drying naturally in air. The heat-initiated chemical reaction was performed at 80 °C in a high-pressure stainless steel container filled with pure nitrogen (Supplementary Fig. S1). Afterwards, the samples were rinsed thoroughly in acetone to remove BPO residues for subsequent Raman characterization. Figure 1 shows the time evolution of the Raman spectra for BPO-functionalized graphene heated at 80 °C for 5 min, 10 min, 20 min, 25 min and 30 min in nitrogen atmosphere, respectively. As the reaction time increased, the characteristic disorder-induced D band around 1330 cm^−1^ emerged and gradually became the most prominent feature of the Raman spectra. The D-band is originated from the A_1g_ mode breathing vibrations of six-membered sp^2^ carbon rings, and becomes Raman active after neighboring sp^2^ carbon atoms are converted to sp^3^ hybridization^9^. In addition, the double resonance 2D band around 2670 cm^−1^ significantly weakened, while the G band around 1580 cm^−1^ was broadened due to the presence of a defect-induced D’ shoulder peak at ~1620 cm^–1^. These observations strongly suggest that covalent C-C bonds were formed and thus a high degree of structural disorder was generated by the transformation from sp^2^ to sp^3^ configuration due to reaction with BPO. Indeed, after 25 min of reaction the Raman spectra looked more like that of graphene oxide or highly disordered carbon-based materials. The Raman signatures of graphene did not change any more after 25 min of reaction. It is probably because BPO molecules adhering directly to graphene were completely decomposed within 25 min, since a second experimental procedure on the functionalized graphene always led to a slight stronger D band (Supplementary Fig. S2).

**Figure 2:**
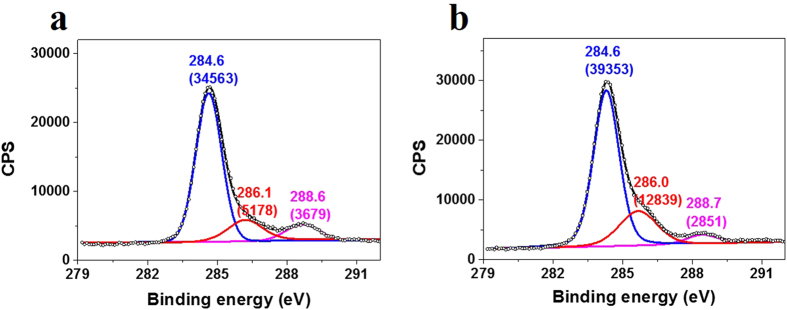
XPS C 1s spectra of (a) pristine graphene and (b) functionalized graphene heated at 80 °C for 25 min in nitrogen atmosphere. Both spectra are fitted with three Lorentzian-Gaussian peaks of 20:80 ratio. The fitted peak positions and areas are indicated on the peaks.

We also did XPS on pristine graphene and functionalized graphene heated at 80 °C for 25 min in nitrogen atmosphere, which could give information about the bond type. Figure 2a,b show the C 1s spectra of pristine graphene and functionalized graphene, respectively (See Supplementary Fig. S3 for survey spectra). Both spectra are fitted with three Lorentzian-Gaussian peaks of 20:80 ratio. The fitted peak positions and areas are indicated on the peaks. The three peaks at 284.6 eV, 286.0 eV and 288.6 eV are attributed to the sp^2^ carbon bonds, sp^3^ carbon bonds and O-C = O bonds, respectively^26^. It can be seen that, the relative area of the peak at 286.0 eV compared to that at 284.6 eV increased after functionalization, indicating an increase in the number and contribution of graphene derived sp^3^ carbon bonds^27^ due to the reaction with BPO. It is also noted that the relative area of the peak at 288.6 eV compared to that at 284.6 eV decreased after functionalization, which indicates two facts: (1) that the immobilized groups are not benzoyloxyl groups but phenyl groups; (2) that the oxygen related defects can react with phenyl radicals and be substituted by phenyl groups. The above XPS analysis not only confirms further the reaction with BPO inferred by Raman spectra, but also excludes benzoyloxyl from the immobilized functional groups.

### Effect of temperature

**Figure 3:**
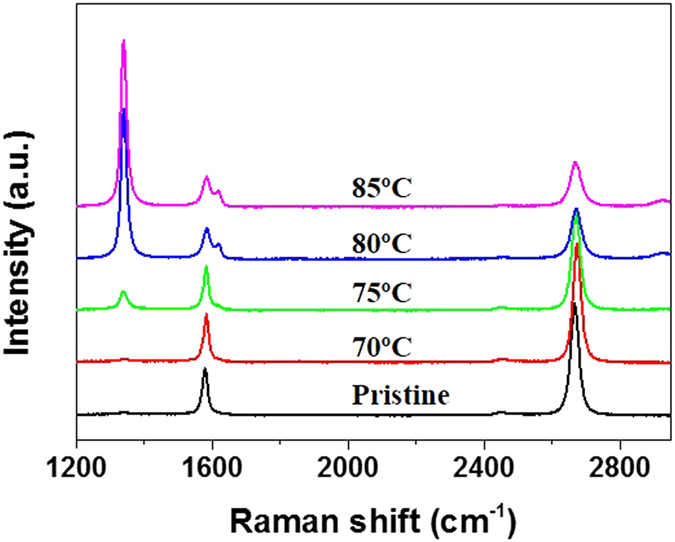
Raman spectra of pristine graphene and Raman spectra of graphene reacted with BPO for 25 min at 70 °C, 75 °C, 80 °C and 85 °C in nitrogen atmosphere, respectively.

The heat-initiated chemical functionalization was also conducted at temperatures below and above 80 °C by changing the preset temperature of the oven. Figure 3 shows the Raman spectra of graphene functionalized for 25 min at 70 °C, 75 °C, 80 °C and 85 °C in nitrogen atmosphere, respectively. It can be seen that no D band could be observed at 70 °C, indicating BPO did not react with graphene at 70 °C. If Raman characterization was done before removing BPO residues, a gradually increasing D band was observed with the Raman laser irradiation time, because the Raman laser could also initiate the reaction^24^. This experiment indicates that at 70 °C BPO did not decompose so that no functionalization occurred. A slight D band appeared when the preset temperature was 75 °C. At 80 °C and 85 °C, a significant D band could be observed, and there is no noticeable differences between the two spectra. Available literature^28^ shows that when benzoyl peroxide was held for prolonged periods at temperatures of 75 °C–80 °C decomposition might occur. Hence it is reasonable that no reaction occurred at 70 °C and a mild functionalization by BPO was observed at 75 °C. Since BPO molecules adhering directly to graphene were completely decomposed within 25 min at 80 °C, the same thing could be expected at 85 °C. It could be further expected that there are no differences in Raman spectra of graphene functionalized at 80 °C and 85 °C because no BPO adhering directly to graphene existed after 25 min at both temperatures.

### Effect of atmosphere

**Figure 4:**
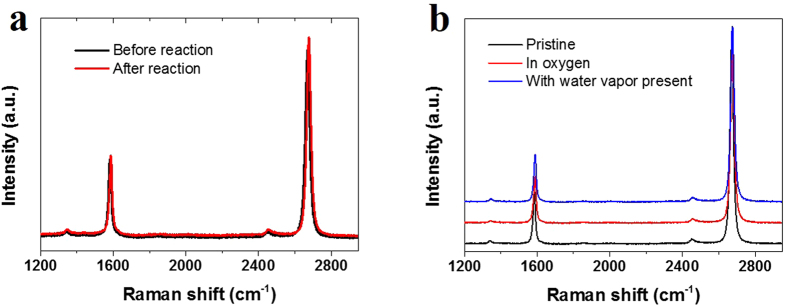
(**a**) Raman spectra of graphene before reaction (black) and after reaction with BPO (red) in air atmosphere. (**b**) Raman spectra of pristine graphene (black) and Raman spectra of graphene reacted with BPO in pure oxygen (red) and in the presence of water vapor (blue).

The heat-initiated chemical functionalization was also conducted in air atmosphere at 80 °C, and the Raman spectra are shown in Fig. 4a. It can be found that no D band could be detected when heating for 25 min. To explore the underlying mechanisms, we investigated the influence of oxygen and water vapor on the reaction, respectively. The influence of oxygen was studied by performing the reaction in the high-pressure stainless steel container filled with pure oxygen at 80 °C for 25 min. Figure 4b shows the Raman spectra of pristine graphene and that reacted with BPO in oxygen-filled container at 85 °C for 25 min. It can be seen that no D band appeared compared to pristine graphene, just as that in air atmosphere. It is widely known that oxygen, which could attach to organic radicals, is an inhibitor in polymerization reactions^29^. The product phenylperoxy radical, which is not as chemically reactive as phenyl radical^29^, is thought to hardly react with inert graphene.

To study the influence of water vapor, a few drops of deionized water was added into the nitrogen-filled container before initiating the decomposition of BPO. Figure 4b shows the Raman spectra of pristine graphene and that reacted with BPO in nitrogen-filled container with water vapor present. It can be seen that no D-band appeared compared to pristine graphene, just as that in air atmosphere. It is speculated that benzoyloxyl radical might accept an electron from water, forming inactive benzoate. BPO was usually stored in water for safety partly for this reason. Actually in air atmosphere water vapor is far less than oxygen, hence it is believed that it is oxygen that mainly inhibits the reaction in air atmosphere.

## Discussion

**Figure 5:**
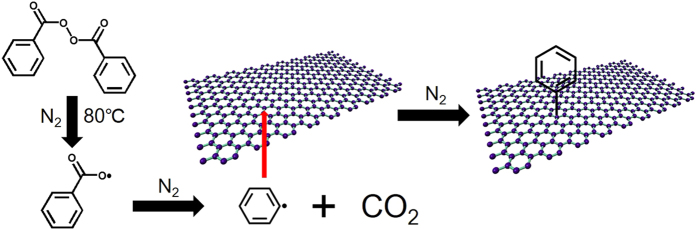
Schematic illustration of proposed direct radical mechanism in heat-initiated chemical functionalization of graphene by BPO.

Based on the above results, a direct radical mechanism was proposed for heat-initiated reaction (Fig. 5), which is different from the indirect radical mechanism in the photon-initiated reaction^24^. As shown in Fig. 5, benzoyloxyl radicals were created by the thermal decomposition of BPO^30^,^31^. Subsequent decarboxylation of the benzoyloxyl radical produced the phenyl radical. In the photon-initiated reaction^24^, excited states in graphene were created following a photon absorbed by graphene. BPO accepted an electron from photoexcited graphene and then decomposed to phenyl radicals. Then in both reactions, phenyl radicals attacked the directly adhering carbon atoms in graphene, resulting in covalent bonds between them. Note that the largest relative intensity of D band compared to G band after heat-initiated reaction is much stronger than that after photon-initiated reaction. One possible reason is that in the solution phase of the photon-initiated reaction, graphene surface is partly covered by BPO molecules due to the dynamic adsorption equilibrium. However, in the heat-initiated solid-phase reaction, the whole graphene surface is covered by BPO molecules after the drying process, so that more BPO molecules are adhering directly onto the graphene surface, resulting in more phenyl groups bonded with graphene. Another possible reason is that in the photon-initiated reaction the photon absorption by graphene would decrease as graphene was functionalized, which would decrease the generation of phenyl radicals and hence the functionalization.

There are a few implications from the heat-initiated chemical reaction. Firstly, it provides a facile method to covalently functionalize graphene at a large-scale and low-cost level. It is a solid-phase reaction, making it more safely and easily be handled by those who are not expert in chemistry. As shown in Figs 1 and 3, the chemical functionalization can be controlled by heating temperature and heating time. The acetone solution of BPO can be used repeatedly, and besides, ethanol and water can also be used as solvent in some cases (not shown), making it environmentally friendly. These advantages make the heat-initiated chemical reaction more favorable than diazonium chemistry^8^,^22^ and photochemistry^7^,^23^,^24^ in the chemical functionalization of graphene at large scale.

**Figure 6:**
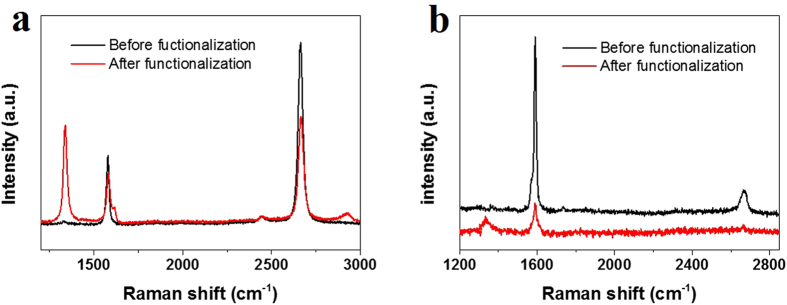
(**a**) Raman spectra of graphene before functionalization (thick black) and after functionalization by laurel peroxide (thin red) at 70 °C for 45 min. (**b**) Raman spectra of an isolated SWNT before functionalization (black) and after functionalization by BPO (red) at 80 °C for 25 min in air atmosphere.

Secondly, it can be extended to other carbon nanomaterials, and other molecules that can be thermally converted to active radicals. Here laurel peroxide and single-walled carbon nanotubes (SWNTs) are taken as examples, respectively. Figure 6a shows the Raman spectra of graphene before reaction and after reaction with laurel peroxide at 70 °C for 45 min. It can be seen that after reaction there was a prominent D band, which was just a little weaker than that after reaction with BPO at 80 °C. It is because significant decomposition of laurel peroxide can occur at 70 °C which is lower than that of BPO. This experiment indicates that dodecyl groups were covalently bonded with graphene.

Figure 6b shows the Raman spectra of an isolated SWNT before reaction and after reaction with BPO at 80 °C for 25 min in air atmosphere. It can be seen that after reaction, an obvious D band appeared around 1330 cm^−1^ and G band weakened a lot, indicating that the SWNT was functionalized by BPO. Note that the functionalization of SWNTs was performed in air atmosphere. It is known that due to the extremely small curvature and hence large strain, SWNTs are not as inert as graphene and hence are expected to react with phenyl radials faster than graphene. The increased reaction rate between phenyl radicals and carbon nanotubes is believed to be higher than that between phenyl and graphene and is speculated to be comparable to that between phenyl radicals and oxygen, leading to a more-likely-occurred functionalization of SWNTs. The above two experiments demonstrate that the heat-initiated chemical reaction is a universal method in chemical functionalization of carbon nanomaterials by various functional groups.

**Figure 7:**
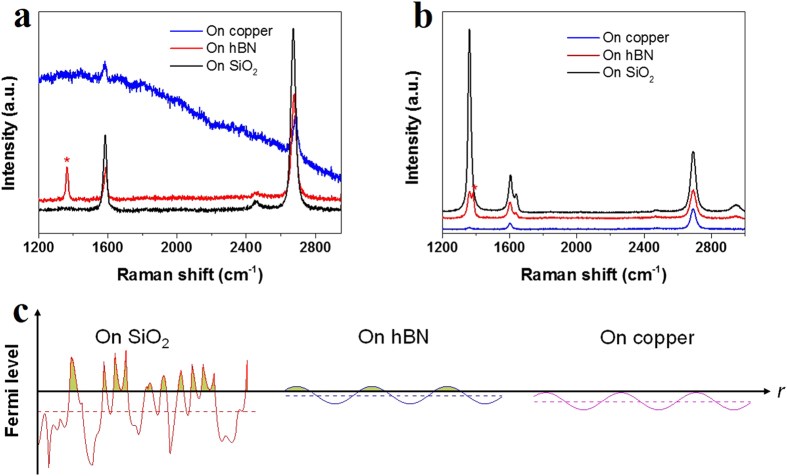
Raman spectra of graphene (a) before and (b) after functionalization with BPO on SiO_2_, copper and hBN. The Raman spectra of graphene after functionalization with BPO on copper were collected after transferring the functionalized graphene onto SiO_2_. The peaks indicated by asterisks are from hBN. (**c**) Various electron-hole charge fluctuations due to the locally n-doped puddles in overall p-doped graphene on SiO_2_, hBN and copper. Solid curves indicate spatial variation of the local Fermi level in electron-hole puddles, and the dashed lines indicate the average Fermi level.

Thirdly, to better utilize the heat-initiated chemical reaction and to tune the chemical functionalization, reactions between BPO and graphene on various substrates were performed. Graphene is a type of materials, in which all atoms are on surface, making their properties to be highly allergic to adsorbed molecules^32^,^33^,^34^ and supported substrates^35^,^36^,^37^. Pristine supported graphene is a semimetal slightly p-doped by oxygen, depending on the substrates^38^. Here CVD-grown graphene on copper was either directly functionalized at 80 °C for 25 min or was transferred onto SiO_2_ and hexagonal boron nitride (hBN) followed by functionalization with BPO at 80 °C for 25 min. Figure 7a shows the Raman spectra of graphene on SiO_2_, copper and hBN substrates before BPO functionalization. The peak indicated by asterisk is from hBN. The integrated intensity ratio between 2D band and G band of graphene on SiO_2_, copper and hBN (I_2D_/I_G_) are 3.62, 4.02 and 5.74, respectively. As reported, I_2D_/I_G_ is an indicator of doping level, where a larger I_2D_/I_G_ corresponds to a lower doping level^38^. It can be inferred that graphene on hBN is least doped, while graphene on copper is more highly doped and the doping of graphene on SiO_2_ is the highest.

Figure 7b shows the Raman spectra of graphene on SiO_2_, copper and hBN substrates after BPO functionalization. For stronger peaks, the Raman spectra of graphene after functionalization on copper were collected after transferring the functionalized graphene onto SiO_2_, which would not change the relative intensities between the peaks. After functionalization a prominent D band appeared on SiO_2_ substrate, indicating the significant formation of sp^3^ bonds. On hBN substrate, a mild D band appeared, indicating a slight covalent functionalization. On copper substrate, a very small D band appeared, meaning a sparse formation of sp^3^ bonds. As shown in Fig. 5, the sp^3^ bonds could form when phenyl radicals attack graphene. Phenyl radical is a highly oxidative species, whose reactivity increases for increasingly n-doped graphene and is negligible for p-doped graphene^39^. Therefore the electronic density of states and Fermi level of graphene directly influence the reaction rate^35^. The schematic illustration in Fig. 7c shows various electron-hole charge fluctuations due to the locally n-doped puddles in overall p-doped graphene on SiO_2_, hBN and copper^35^,^40^,^41^. Although graphene on SiO_2_ is the most p-doped, large electron and hole fluctuations^41^ in graphene can be induced by charged impurities and polar adsorbates on SiO_2_ surface, resulting in the reaction to likely occur. Graphene on hBN is the least p-doped but has small electron-hole fluctuations due to its atomic flatness within large area^42^, and hence has lower reactivity. For moderately p-doped graphene grown on copper, graphene is closely adhering to clean copper at atomic level, so that the local electron-hole fluctuation is speculated at the similar level as that on hBN. Hence it is hardly possible for the graphene on copper to donate electrons and form sp^3^ bonds.

**Figure 8:**
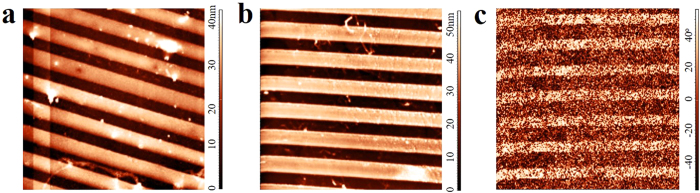
(**a**) AFM height image of BPO pattern on graphene (**b**) AFM height image of BPO residues pattern on graphene before rinsing. (**c**) AFM phase images of functionalized graphene after rinsing out BPO remains. The sizes of all AFM images are 10 μm × 10 μm.

Finally, graphene pattern can be prepared by combing heat-initiated chemical functionalization and microfluidic technique. Polydimethylsiloxane (PDMS) stamp, which was prepared from compact disk (CD), was gently pressed against graphene sample. BPO solution was led into the tunnels between PDMS stamp and graphene. After drying, graphene was separated from PDMS to obtain BPO pattern on graphene (Supplementary Fig. S4). Chemical reaction was thermally initiated to obtain graphene pattern. Figure 8a shows the AFM height image of BPO pattern on graphene and Fig. 8b shows the AFM height image of BPO residues pattern on graphene before rinsing. Well-aligned stripes can be seen in both images, which are consistent with the periodical structure in CD. Since the pattern height is not uniform at large scale, we cannot compare the pattern height in Figs. 8a-8b. Fig. 8c shows the AFM phase image of functionalized graphene after rinsing out BPO residues. The functionalized graphene stripes cannot be clearly identified in the height image due to the relatively small height of the lying down phenyl group (not shown). The phase image gives well-aligned stripes because of different surface properties between functionalized and pristine graphene. It is demonstrated that this method provides a facile way to fabricate large-scale graphene patterns, which avoids the strong photoresist adhesion to graphene in photolithography.

In conclusion, a heat-initiated chemical reaction was developed to functionalize CVD-grown graphene at wafer scale and the reaction was universally extended to carbon nanotubes, and other precursors that can be thermally converted to active radicals. The chemical reaction could occur in absence of oxygen and water vapor when the temperature is above the decomposition temperature of the reactants. The chemical reaction was also found to be substrate-dependent due to surface doping and inhomogeneity. A large-scale graphene pattern was demonstrated by combing with microfluidic technique. This heat-initiated solid-phase chemical reaction provide a facile and environmentally friendly approach to functionalize carbon nanomaterials with various functional groups.

## Methods

### Preparation of single-layer graphene samples

Single-layer graphene was grown on copper foil at temperature of 1000 °C by low-pressure CVD using a mixture of 0.15 sccm CH_4_ and 4.00 sccm H_2_ at a total pressure of ~100 mTorr. For subsequent characterizations and functionalization, the CVD-grown graphene was transferred onto other substrates as follows. PMMA solution (M_w_ = 950 K, 4 wt%) was spin-coated onto the graphene-covered copper foil at 3000 rpm for 1 min and then baked at 100 °C for 30 min to form a ~250 nm-thick thin film The PMMA film was detached by etching the copper away with 2.0 M FeCl_3_ aqueous solution and then attached to silicon substrate with 300 nm thick silicon oxide (SiO_2_) and hBN flakes. Finally, PMMA film was dissolved in hot acetone, leaving the transferred graphene anchored on target substrates.

### Preparation of thin hBN flakes

Thin hBN flakes were prepared by micromechanical cleavage of commercially available hBN crystals (Momentive, Polartherm grade PT110). The crystals have an average size of ~45 μm. A tiny amount of hBN powder was placed on a stripe of scotch tape, which was then repeatedly folded onto itself. Subsequently, a SiO_2_ substrate was pressed onto the tape. After peeling off the tape, a few hBN flakes could be left on the SiO_2_ surface.

### Heat-initiated chemical functionalization of graphene

The graphene samples were immersed into 10 mM reactants’ (i.e. BPO and laurel peroxide) acetone solution, and were then taken out drying for chemical reaction. The heat-initiated chemical reaction was carried out in a high-pressure stainless-steel container with the outlet connecting with a pump and the inlet connecting with a nitrogen cylinder. To this end, the graphene samples were placed into the container, followed by sealing with a flange cover. The container was filled with pure nitrogen by repeating pumping and inflating with nitrogen 5 times. The container was then put into a preheated oven to initiate the reaction. Afterwards, the graphene samples were rinsed thoroughly in acetone to remove BPO residues for subsequent characterization.

### Characterization

Raman spectroscopy (B&W-Tek) with laser excitation energy of 532 nm (2.33 eV) was used to evaluate the graphene quality and the functionalization results. The spectra were recorded with an acquisition time of 1 s by averaging 10 scans over the range 1200–3000 cm^−1^. The laser spot size is ~1 μm. The spectral resolution is ~2 cm^−1^. The functionalized graphene on copper was transferred onto SiO_2_ substrate for enhanced Raman signals. Atomic force microscope (NT-MDT) in tapping mode was used for height and phase imaging of graphene pattern and SWNTs. XPS spectra were recorded using a Thermo Scientific K-Alpha spectrometer at pressures lower than 5 × 10^−9^ mBar.

### Preparation of SWNTs sample

Highly oriented SWNTs with lengths from tens to hundreds of microns were grown on single crystal ST-cut quartz (Hoffman Materials Inc.). The 0.3 mM CoCl_2_ alcoholic solution, used as the catalyst, was spin-coated onto quartz surface. The growth was performed at 870 °C with a flow of methane (1000 sccm) and hydrogen (140 sccm) for 30 min. SWNTs were then transferred onto a SiO_2_ substrate with markers (Supplementary Fig. S5) for locating isolated SWNTs in Raman characterization.

### Fabrication of graphene pattern

Graphene pattern was fabricated by combining heat-initiated chemical reaction and microfluidic technique. PDMS stamps were prepared by using inexpensive commercially available CD, where the polycarbonate surface was used as masters after peeling off aluminum layer with tweezers. A bubble-free mixture of elastomer and curing agent with the ratio of 5:1 was poured onto the polycarbonate surface and was then baked at 120 °C for 60 min. After peeling off the master, cured PDMS stamp was available for subsequent microfluidic experiment. To this end, PDMS stamp was pressed against the graphene sample, and slowly put into BPO solution with one end of tunnels in the solution and the other end above the solution. After 5 min, the assembly was taken out and was dried overnight. After separating the PDMS stamp, patterned BPO molecules on graphene were obtained for subsequent heat-initiated chemical reaction.

## Additional Information

**How to cite this article**: Gao, G. *et al.* Heat-Initiated Chemical Functionalization of Graphene. *Sci. Rep.* **6**, 20034; doi: 10.1038/srep20034 (2016).

## Supplementary Material

Supplementary Information
