# Supplementary material for: Heat-Initiated Chemical Functionalization of Graphene
Source: Sci Rep. 2016 Jan 28;6:20034. doi: 10.1038/srep20034 (PMC4730243; doi:10.1038/srep20034)
Supplement: Supplementary Information [file srep20034-s1.pdf]

Heat-Initiated Chemical Functionalization of Graphene

Guodong Gao[1], Dandan Liu[1], Shangcheng Tang[1], Can Huang[1], Mengci He[1], Yu Guo[2] Xiudong Sun[1] and Bo Gao[1]

[1]Institute of Modern Optics, Key Lab of Micro-optics and Photonic Technology of Heilongjiang Province, Department of Physics, Harbin Institute of Technology, Harbin 150001, China.

[2]School of Materials Science and Technology, Harbin Institute of Technology, Harbin 150001, China.

Correspondence and requests for materials should be addressed to Bo Gao (email: gaobo@hit.edu.cn).

1. Schematic figure and photograph of high-pressure stainless steel container

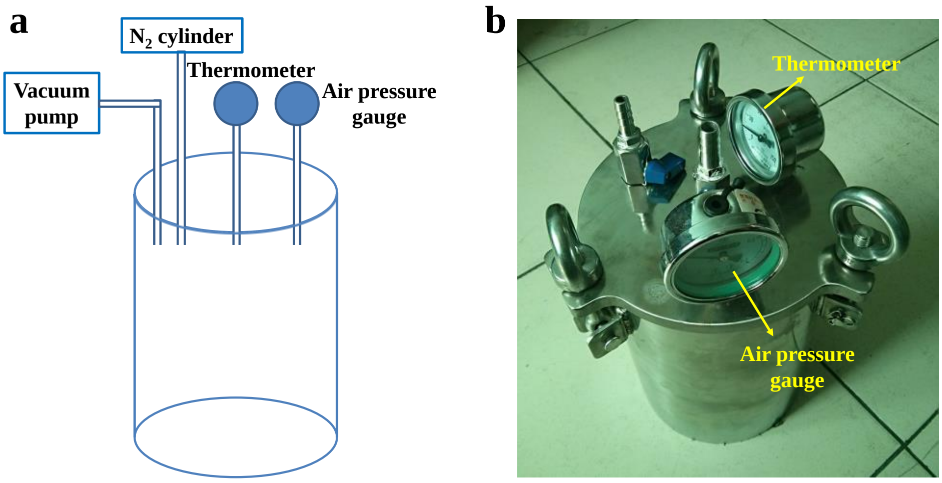

Figure S1. (a) Schematic figure and (b) photograph of high-pressure stainless steel container.

2. Raman spectra of graphene functionalized after the first reaction and after a second reaction

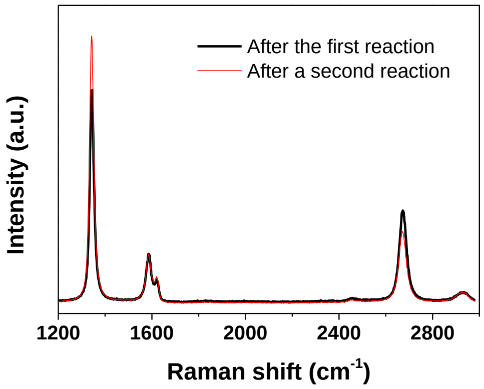

Figure S2. Raman spectra of graphene functionalized after the first reaction (thick black line) and after a second reaction (thin red line).

3. XPS survey spectra of pristine and functionalized graphene

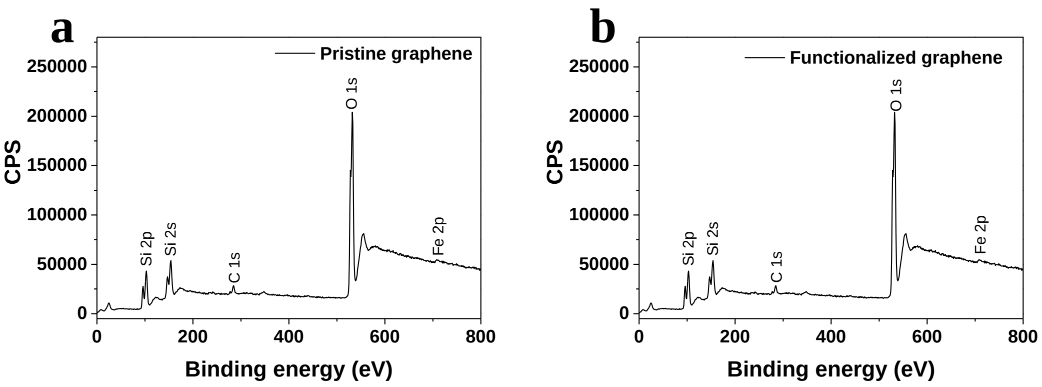

Figure S3 XPS survey spectra of (a) pristine and (b) functionalized graphene.

4. Optical image of large-scale BPO pattern on graphene

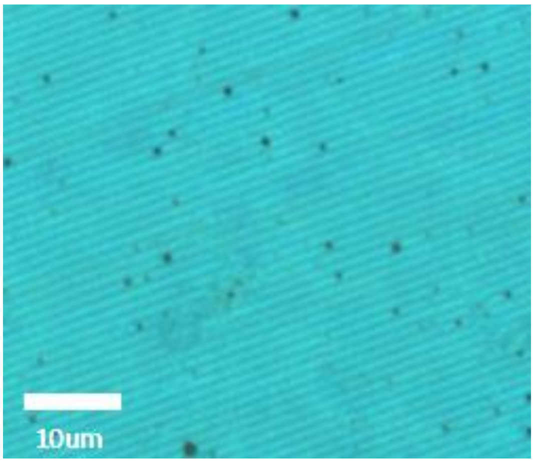

Figure S4. Optical image of large-scale BPO pattern on graphene. The scale bar is 10 μm.

5. SWNTs sample

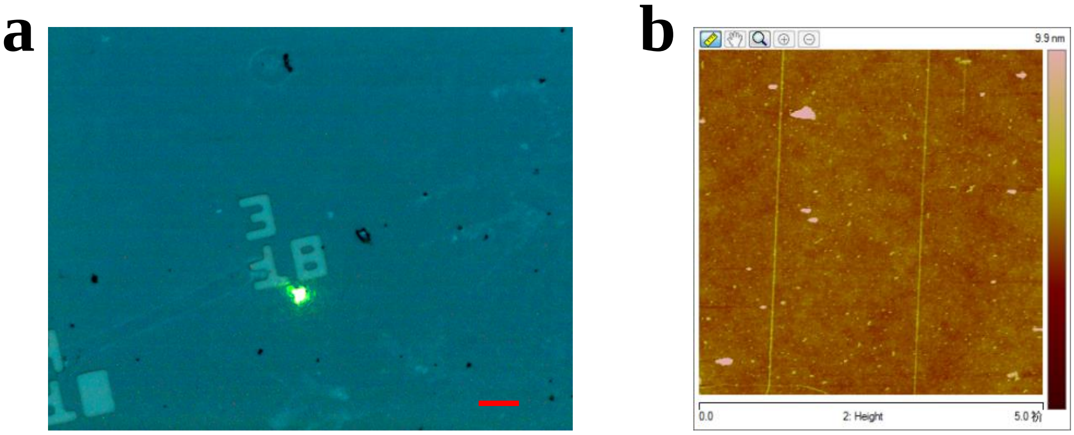

Figure S5. (a) Optical image of SiO_2 substrate with markers. The bright laser spot is where the SWNT located. The scale bar is 10 μm. (b) Typical AFM height image of horizontally aligned isolated SWNTs on SiO_2 (not the SWNT in a). The AFM image size is 5 μm x 5 μm.
